# Impact of HbA1c Measurement on Hospital Readmission Rates: Analysis of 70,000 Clinical Database Patient Records

**DOI:** 10.1155/2014/781670

**Published:** 2014-04-03

**Authors:** Beata Strack, Jonathan P. DeShazo, Chris Gennings, Juan L. Olmo, Sebastian Ventura, Krzysztof J. Cios, John N. Clore

**Affiliations:** ^1^Department of Computer Science, Virginia Commonwealth University, Richmond, VA 23284, USA; ^2^Department of Health Administration, Virginia Commonwealth University, Richmond, VA 23298, USA; ^3^Department of Biostatistics, Virginia Commonwealth University, Richmond, VA 23298, USA; ^4^Department of Computer Science and Numerical Analysis, University of Cordoba, 14071 Cordoba, Spain; ^5^IITiS Polish Academy of Sciences, 44-100 Gliwice, Poland; ^6^Department of Medicine, Virginia Commonwealth University, Richmond, VA 23298, USA

## Abstract

Management of hyperglycemia in hospitalized patients has a significant bearing on outcome, in terms of both morbidity and mortality. However, there are few national assessments of diabetes care during hospitalization which could serve as a baseline for change. This analysis of a large clinical database (74 million unique encounters corresponding to 17 million unique patients) was undertaken to provide such an assessment and to find future directions which might lead to improvements in patient safety. Almost 70,000 inpatient diabetes encounters were identified with sufficient detail for analysis. Multivariable logistic regression was used to fit the relationship between the measurement of HbA1c and early readmission while controlling for covariates such as demographics, severity and type of the disease, and type of admission. Results show that the measurement of HbA1c was performed infrequently (18.4%) in the inpatient setting. The statistical model suggests that the relationship between the probability of readmission and the HbA1c measurement depends on the primary diagnosis. The data suggest further that the greater attention to diabetes reflected in HbA1c determination may improve patient outcomes and lower cost of inpatient care.

## 1. Introduction

It is increasingly recognized that the management of hyperglycemia in the hospitalized patient has a significant bearing on outcome, in terms of both morbidity and mortality [1, 2]. This recognition has led to the development of formalized protocols in the intensive care unit (ICU) setting with rigorous glucose targets in many institutions [3]. However, the same cannot be said for most non-ICU inpatient admissions. Rather, anecdotal evidence suggests that inpatient management is arbitrary and often leads to either no treatment at all or wide fluctuations in glucose when traditional management strategies are employed. Although data are few, recent controlled trials have demonstrated that protocol-driven inpatient strategies can be both effective and safe [4, 5]. As such, implementation of protocols in the hospital setting is now recommended [6, 7]. However, there are few national assessments of diabetes care in the hospitalized patient which could serve as a baseline for change. The present analysis of a large clinical database was undertaken to examine historical patterns of diabetes care in patients with diabetes admitted to a US hospital and to inform future directions which might lead to improvements in patient safety. In particular, we examined the use of HbA1c as a marker of attention to diabetes care in a large number of individuals identified as having a diagnosis of diabetes mellitus. We hypothesize that measurement of HbA1c is associated with a reduction in readmission rates in individuals admitted to the hospital.

Databases of clinical data contain valuable but heterogeneous and difficult data in terms of missing values, incomplete or inconsistent records, and high dimensionality understood not only by number of features but also their complexity. [8]. Additionally, analyzing external data is more challenging than analysis of results of a carefully designed experiment or trial, because one has no impact on how and what type of information was collected. Nonetheless, it is important to utilize these huge amounts of data to find new information/knowledge that is possibly not available anywhere.

## 2. Materials and Methods

### 2.1. Data Assembly

This study used the Health Facts database (Cerner Corporation, Kansas City, MO), a national data warehouse that collects comprehensive clinical records across hospitals throughout the United States. Health Facts is a voluntary program offered to organizations which use the Cerner Electronic Health Record System. The database contains data systematically collected from participating institutions electronic medical records and includes encounter data (emergency, outpatient, and inpatient), provider specialty, demographics (age, sex, and race), diagnoses and in-hospital procedures documented by ICD-9-CM codes, laboratory data, pharmacy data, in-hospital mortality, and hospital characteristics. All data were deidentified in compliance with the Health Insurance Portability and Accountability Act of 1996 before being provided to the investigators. Continuity of patient encounters within the same health system (EHR system) is preserved.

The Health Facts data we used was an extract representing 10 years (1999–2008) of clinical care at 130 hospitals and integrated delivery networks throughout the United States: Midwest (18 hospitals), Northeast (58), South (28), and West (16). Most of the hospitals (78) have bed size between 100 and 499, 38 hospitals have bed size less than 100, and bed size of 14 hospitals is greater than 500.

The database consists of 41 tables in a fact-dimension schema and a total of 117 features. The database includes 74,036,643 unique encounters (visits) that correspond to 17,880,231 unique patients and 2,889,571 providers. Because this data represents integrated delivery network health systems in addition to stand-alone hospitals, the data contains both inpatient and outpatient data, including emergency department, for the same group of patients. However, data from out-of-network providers is not captured.

The dataset was created in two steps. First, encounters of interest were extracted from the database with 55 attributes. This dataset is available as a Supplementary Material available online at http://dx.doi.org/10.1155/2014/781670 and is also in the process of submission to the UCI Machine Learning Repository [9] so that it is easily available to other researchers.

Second, preliminary analysis and preprocessing of the data were performed resulting in retaining only these features (attributes) and encounters that could be used in further analysis, that is, contain sufficient information. Both steps are described in the following subsections.

### 2.2. Extraction of the Initial Dataset from the Database

Information was extracted from the database for encounters that satisfied the following criteria.

1. It is an inpatient encounter (a hospital admission).
2. It is a “diabetic” encounter, that is, one during which any kind of diabetes was entered to the system as a diagnosis.
3. The length of stay was at least 1 day and at most 14 days.
4. Laboratory tests were performed during the encounter.
5. Medications were administered during the encounter.

Criteria 3-4 were applied to remove admissions for procedures and so forth, which were of less than 23 hours of duration and in which changes in diabetes management were less likely to have occurred. It should be noted that the diabetic encounters are not all encounters of diabetic patients but rather only these encounters where diabetes was coded as an existing health condition.

101,766 encounters were identified to fulfill all of the above five inclusion criteria and were used in further analysis. Attribute/feature selection was performed by our clinical experts and only attributes that were potentially associated with the diabetic condition or management were retained. From the information available in the database, we extracted 55 features describing the diabetic encounters, including demographics, diagnoses, diabetic medications, number of visits in the year preceding the encounter, and payer information. The full list of the features and their description is provided in Table 1.

Since we are primarily interested in factors that lead to early readmission, we defined the readmission attribute (outcome) as having two values: “readmitted,” if the patient was readmitted within 30 days of discharge or “otherwise,” which covers both readmission after 30 days and no readmission at all. The values of the readmission attribute were determined by examination of all patient records in the database to determine the first inpatient visit after discharge. Note that 30 days was chosen based on criteria often used by funding agencies. Hemoglobin A1c (HbA1c) is an important measure of glucose control, which is widely applied to measure performance of diabetes care [10, 11]. The measurement of HbA1c at the time of hospital admission offers a unique opportunity to assess the efficacy of current therapy and to make changes in that therapy if indicated (e.g., HbA1c > 8.0% on current regimen). We considered the possibility that if an HbA1c test result was available from a measurement (outpatient or inpatient) done within three months prior to the sentinel admission, the test might not be repeated. In these cases (0.1% of the total), we used the measurement available from the previous visit. In all other cases, measurement of HbA1c was performed at the time of hospital admission. We examined both the frequency of HbA1c test ordering and the response to its result, which we defined as a change in diabetic medications. By a “change of medication” we understand any dosage change (increase or reduction) as well as change to a drug with a different generic name, for example, a change of the type of insulin or an introduction of a new drug. The database contains detailed information about dosage but is restricted only to medications administered during the encounter. It was not possible to track any preadmission and discharge medications.

We considered four groups of encounters: (1) no HbA1c test performed, (2) HbA1c performed and in normal range, (3) HbA1c performed and the result is greater than 8% with no change in diabetic medications, and (4) HbA1c performed, result is greater than 8%, and diabetic medication was changed.

### 2.3. Preliminary Analysis and the Final Dataset

The original database contains incomplete, redundant, and noisy information as expected in any real-world data. There were several features that could not be treated directly since they had a high percentage of missing values. These features were weight (97% values missing), payer code (40%), and medical specialty (47%). Weight attribute was considered to be too sparse and it was not included in further analysis. Payer code was removed since it had a high percentage of missing values and it was not considered relevant to the outcome. Medical specialty attribute was maintained, adding the value “missing” in order to account for missing values. Large percentage of missing values of the weight attribute can be explained by the fact that prior to the HITECH legislation of the American Reinvestment and Recovery Act in 2009 hospitals and clinics were not required to capture it in a structured format.

The preliminary dataset contained multiple inpatient visits for some patients and the observations could not be considered as statistically independent, an assumption of the logistic regression model. We thus used only one encounter per patient; in particular, we considered only the first encounter for each patient as the primary admission and determined whether or not they were readmitted within 30 days. Additionally, we removed all encounters that resulted in either discharge to a hospice or patient death, to avoid biasing our analysis. After performing the above-described operations, we were left with 69,984 encounters that constituted the final dataset for analysis.

The variables chosen to control for patient demographic and illness severity were gender, age, race, admission source, discharge disposition, primary diagnosis (see Table 2), medical specialty of the admitting physician, and time spent in hospital. Values of these variables and their distribution in the dataset are shown in Table 3.

To summarize, our dataset consists of hospital admissions of length between one and 14 days that did not result in a patient death or discharge to a hospice. Each encounter corresponds to a unique patient diagnosed with diabetes, although the primary diagnosis may be different. During each of the analyzed encounters, lab tests were ordered and medication was administered.

### 2.4. Statistical Methods

The unit of our analysis is an encounter; however, in order to keep the observations independent, we only analyzed one encounter per patient. After preliminary analysis and taking into account the amount of data, the significance level was determined by a *P* value of less than 0.01.

Multivariable logistic regression was used to fit the relationship between the measurement of HbA1c and early readmission while controlling for covariates such as demographics, severity and type of the disease, and type of admission.

To assess whether the candidate covariates were significantly associated with readmission, we created the model in four steps. Each step was followed by tests for significance of variables with higher degree of freedom, an analysis of deviance table, and sensitivity analysis which was done by removing one variable at the time and looking at changes of beta-coefficients.

First, we fitted a logistic model with all variables but HbA1c. We refer to this model as the core model. Second, we added HbA1c to the core model. Third, we added pairwise interactions to the core model (without HbA1c) and kept only the significant ones. Finally, we added pairwise interactions with HbA1c, leaving only the significant ones in the final model.

Graphics were used to help in the interpretation of interaction terms in the final model. The analysis was performed in R statistical software.

### 2.5. Ethical and Legal Issues

This research is based on a preexisting HIPAA compliant dataset that contains no personally identifiable information. Due to the deidentified nature of the datasets obtained, this study was not considered human subjects research nor required consent per the Helsinki Declaration and was therefore exempt from VCU Institutional Review Board review.

## 3. Results and Discussion

As shown in Table 3, measurement of HbA1c was infrequent, occurring in only 18.4% of encounters where diabetes mellitus was included as an admission diagnosis. Of those in whom the test was ordered, 51.4% were less than 8%. When an HbA1c was not obtained, 42.5% of patients had a medication change during the hospitalization, whereas those providers who ordered the test appear to have been somewhat more responsive as determined by changes in medication (55.0%, *P* < 0.001). Of those in whom the test was ordered and found to be greater than 8%, 65.0% had a documented medication change. With respect to readmission and taken as a whole without adjusting for covariates, measurement of HbA1c was associated with a significantly reduced rate of readmission (9.4 versus 8.7%, *P* = 0.007). This was true regardless of the outcome of the test. We then examined the relationship between readmission and HbA1c adjusting for covariates such as patient demographic and illness type and severity.

Since the gender variable was not significant (*P* = 0.36) in the core model (without HbA1c), it was removed from further analysis. When tested for sensitivity, the values of beta-coefficients in the model changed by less than 35%, with an exception of the time in the hospital, medical specialty, age, and primary diagnosis that changed by 77%, 47%, 49%, and 65%, respectively, when the discharge disposition was removed. This suggests a relationship between these variables.

The significant pairwise interactions between the covariates were discharge disposition with race (*P* < 0.001), medical specialty of the admitting physician (*P* = 0.001), primary diagnosis (*P* = 0.005), and time in hospital (*P* < 0.001); the specialty of the admitting physician with time in hospital (*P* = 0.001) and age (*P* < 0.001); and the primary diagnosis with time in the hospital (*P* < 0.001) and HbA1c (*P* = 0.004). Only these interactions were included in the final model.

**Figure 1:**
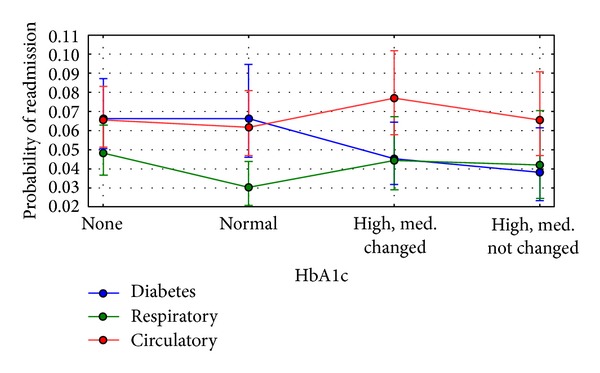
Adjusted for covariates readmission rates by the primary diagnosis and HbA1c measurement. Blue denotes diabetes (icd9: 250.xx), green denotes diseases of the respiratory system (icd9: 460–519, 786), and red denotes diseases of the circulatory system (icd9: 390–459, 785). Readmission rates were predicted on the reference values of other predictors and the mean value of time in hospital (Table 3). The error bars represent the 95% confidence intervals for the predicted values. Three-degree-of-freedom tests show that the profile of readmission in the group with the primary diagnosis of diabetes is different than the primary diagnoses being circulatory (significant, *P* < 0.001) or respiratory (borderline significant, *P* = 0.02) conditions.

**Figure 3:**
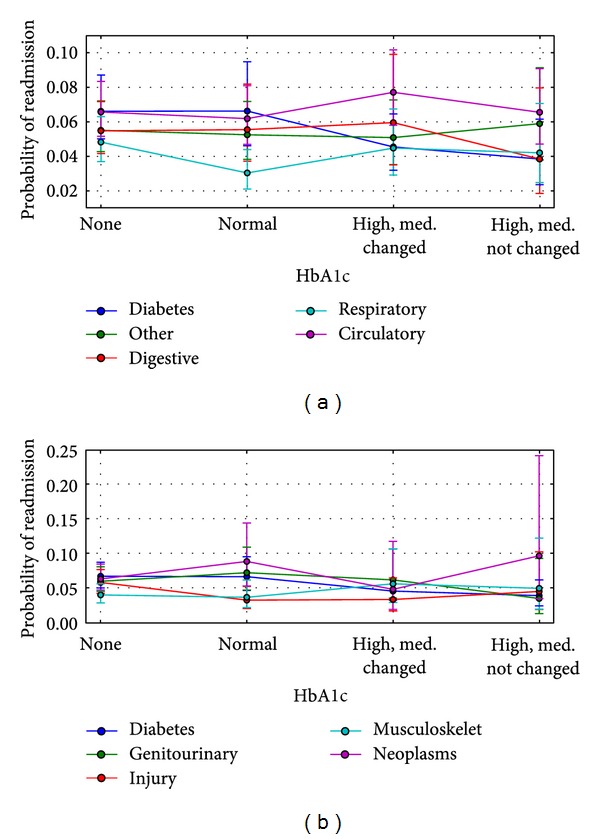
Predicted (adjusted for covariates) readmission rates by the primary diagnosis and HbA1c measurement. Readmission rates were predicted on the reference values of other predictors and the mean value of time in hospital (Table 3). The error bars represent the 95% confidence intervals for the predicted values. The following abbreviations are used for particular icd9 codes: “circulatory” for icd9: 390–459, 785, “digestive” for icd9: 520–579, 787, “genitourinary” for icd9: 580–629, 788, “diabetes” for icd9: 250.xx, “injury” for icd9: 800–999, “musculoskeletal” for icd9: 710–739, “neoplasms” for icd9: 140–239, “respiratory” for icd9: 460–519, 786, and “other” for otherwise.

The final model (Tables 4 and 5) suggests that the relationship between the probability of readmission and the HbA1c measurement significantly depends on the primary diagnosis (note that diabetes is always one of the secondary diagnoses). Specifically, the profile of readmission of patients with a primary diagnosis of diabetes mellitus, after adjusting for covariates, differs significantly from those with a primary diagnosis of circulatory diseases (*P* < 0.001) and approaches significance for those with a primary diagnosis of respiratory diseases (*P* = 0.02).  Figure 1 shows predicted (adjusted for covariates) readmission rates for these three conditions which accounted for 52.4% of all encounters. The predictions were calculated with the mean value of the time in hospital and at reference levels of other covariates. There was no significant interaction with other primary diagnoses (see Figure 3).

The present study provides a striking cross-sectional view of inpatient diabetes care for more than 70,000 admissions in 54 hospitals in the USA. We have designed our analysis using highly conservative criteria. Out of a total of 5 million inpatient admissions in the database, only about 500,000 encounters (just under 10%) were clearly documented as occurring in individuals with diabetes and only almost 70,000 satisfied our inclusion criteria. This is certainly an underestimate given the widespread lack of designation of diabetes mellitus in hospital discharges [12] as well as the prevalence in the USA [13]. Nevertheless, the database permitted us to examine clinical practice over a 10-year period of over 5,000 providers.

First and foremost, the data indicate that, despite widespread recognition of the utility of HbA1c as a performance measure of diabetes care [14, 15], the test is ordered infrequently (18.4%) in the inpatient setting even when test results within the previous 3 months are included (0.1% of the total). It is possible that HbA1c values not in our dataset were available to the practitioners and influenced treatment patterns. However, unlikely, this could be the result of a dual charting system where diagnosis was stored in the electronic health record but these specific laboratory results were not. We recognize this as a potential limitation to our interpretation of the data. But similar analyses by others have confirmed a low rate of HbA1c determinations [16]. We were also surprised at the apparent reluctance of providers to make changes in antihyperglycemic medications during hospitalizations. It should be pointed out that the data considered span a 10-year period (1999–2008). Recommended standards of care which encourage discontinuation of medications on admission and might prompt changes in medications based on glucose control were only recently adopted [17]. When an HbA1c was not obtained, less than half of patients (42.5%) had a medication change during the hospitalization, whereas those providers who ordered the test appear to have been somewhat more responsive to the data as determined by changes in medication (55.0%, *P* < 0.001). Unfortunately, we are not able to determine what drove the medication changes by providers in those patients in whom an HbA1c was not obtained but persistently elevated glucose readings may well explain the practice. It is of interest that a recent analysis of 1274 patients with diabetes admitted for acute myocardial infarction demonstrated only a 31% rate of glucose therapy intensification when a clinical HbA1c result was available [18]. With respect to readmission rate, our data suggest that, regardless of the result, simply measuring HbA1c is associated with a lower rate of readmission in individuals with a primary diagnosis of diabetes mellitus, whereas those with the frequently observed primary diagnoses of circulatory or respiratory diseases are not. It may not be surprising that the attention given to diabetes care in individuals with admitting diagnoses of circulatory or respiratory diseases may have been less than those with a primary diagnosis of diabetes mellitus. However, our findings strongly suggest that greater attention to diabetes care during the hospitalization for these high-risk individuals may have a significant impact on readmission. Our analysis cannot address cause and effect, but the data provide strong support for development of protocols to examine this hypothesis directly. Hospitalization is a unique opportunity for providers to influence change to patient's health outcome trajectories. Resources available in the inpatient setting are often much greater than those available to practitioners in the outpatient setting and could be leveraged to impact care. On average, inpatient stays in the present dataset were 4.27 days which would permit examination of diabetes care and development of a plan for change should it be warranted. The importance of this is emphasized by the readmission data provided.

We recognize that the results from the present analysis represent a preliminary observation with limitations intrinsic to such large health records. In addition to the limitations of working with large clinical datasets discussed earlier, this study is also limited by a nonrandomized study design. Nevertheless, our data appear to support the contention that greater attention to glucose homeostasis during hospital admission may be warranted.

## 4. Conclusions

In conclusion, the decision to obtain a measurement of HbA1c for patients with diabetes mellitus is a useful predictor of readmission rates which may prove valuable in the development of strategies to reduce readmission rates and costs for the care of individuals with diabetes mellitus. For instance, our analysis showed that the profile of readmission differed significantly in patients where Hba1c was checked in the setting of a primary diabetes diagnosis, when compared to those with a primary circulatory disorder. While readmission rates remained the highest for patients with circulatory diagnoses, readmission rates for patients with diabetes appeared to be associated with the decision to test for HbA1c, rather than the values of the HbA1c result.

## Supplementary Material

The Supplemental Materials consist of the dataset as described in Sections 2.1 and 2.2 and in Table 1. The dataset is in csv format. The additional file provides mappings for some of the features.

## Figures and Tables

**Figure 2:**
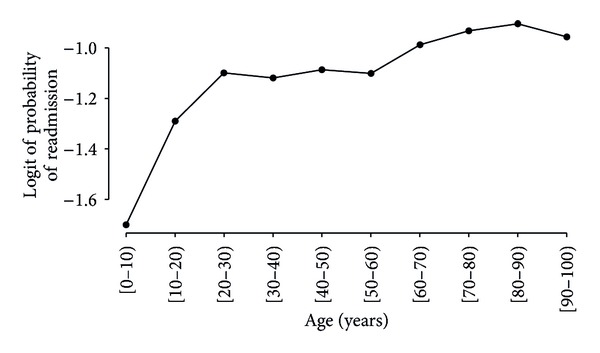
The relationship of age (grouped into intervals of 10 years) and the logistic function of the readmission rate. One can notice that there are three distinct intervals ([0, 30), [30, 60), and [60, 100)) where the relationship has noticeably distinct behavior. This preliminary plot was the motivation to divide the age variable into three categories (Table 3).

**Table 1:** List of features and their descriptions in the initial dataset (the dataset is also available at the website of Data Mining and Biomedical Informatics Lab at VCU (http://www.cioslab.vcu.edu/)).

Feature name	Type	Description and values	% missing
Encounter ID	Numeric	Unique identifier of an encounter	0%
Patient number	Numeric	Unique identifier of a patient	0%
Race	Nominal	Values: Caucasian, Asian, African American, Hispanic, and other	2%
Gender	Nominal	Values: male, female, and unknown/invalid	0%
Age	Nominal	Grouped in 10-year intervals: [0, 10), [10, 20),…, [90, 100)	0%
Weight	Numeric	Weight in pounds.	97%
Admission type	Nominal	Integer identifier corresponding to 9 distinct values, for example, emergency, urgent, elective, newborn, and not available	0%
Discharge disposition	Nominal	Integer identifier corresponding to 29 distinct values, for example, discharged to home, expired, and not available	0%
Admission source	Nominal	Integer identifier corresponding to 21 distinct values, for example, physician referral, emergency room, and transfer from a hospital	0%
Time in hospital	Numeric	Integer number of days between admission and discharge	0%
Payer code	Nominal	Integer identifier corresponding to 23 distinct values, for example, Blue Cross*/*Blue Shield, Medicare, and self-pay	52%
Medical specialty	Nominal	Integer identifier of a specialty of the admitting physician, corresponding to 84 distinct values, for example, cardiology, internal medicine, family*/*general practice, and surgeon	53%
Number of lab procedures	Numeric	Number of lab tests performed during the encounter	0%
Number of procedures	Numeric	Number of procedures (other than lab tests) performed during the encounter	0%
Number of medications	Numeric	Number of distinct generic names administered during the encounter	0%
Number of outpatient visits	Numeric	Number of outpatient visits of the patient in the year preceding the encounter	0%
Number of emergency visits	Numeric	Number of emergency visits of the patient in the year preceding the encounter	0%
Number of inpatient visits	Numeric	Number of inpatient visits of the patient in the year preceding the encounter	0%
Diagnosis 1	Nominal	The primary diagnosis (coded as first three digits of ICD9); 848 distinct values	0%
Diagnosis 2	Nominal	Secondary diagnosis (coded as first three digits of ICD9); 923 distinct values	0%
Diagnosis 3	Nominal	Additional secondary diagnosis (coded as first three digits of ICD9); 954 distinct values	1%
Number of diagnoses	Numeric	Number of diagnoses entered to the system	0%
Glucose serum test result	Nominal	Indicates the range of the result or if the test was not taken. Values: “>200,” “>300,” “normal,” and “none” if not measured	0%
A1c test result	Nominal	Indicates the range of the result or if the test was not taken. Values: “>8” if the result was greater than 8%, “>7” if the result was greater than 7% but less than 8%, “normal” if the result was less than 7%, and “none” if not measured.	0%
Change of medications	Nominal	Indicates if there was a change in diabetic medications (either dosage or generic name). Values: “change” and “no change”	0%
Diabetes medications	Nominal	Indicates if there was any diabetic medication prescribed. Values: “yes” and “no”	0%
24 features for medications	Nominal	For the generic names: metformin, repaglinide, nateglinide, chlorpropamide, glimepiride, acetohexamide, glipizide, glyburide, tolbutamide, pioglitazone, rosiglitazone, acarbose, miglitol, troglitazone, tolazamide, examide, sitagliptin, insulin, glyburide-metformin, glipizide-metformin, glimepiride-pioglitazone, metformin-rosiglitazone, and metformin-pioglitazone, the feature indicates whether the drug was prescribed or there was a change in the dosage. Values: “up” if the dosage was increased during the encounter, “down” if the dosage was decreased, “steady” if the dosage did not change, and “no” if the drug was not prescribed	0%
Readmitted	Nominal	Days to inpatient readmission. Values: “<30” if the patient was readmitted in less than 30 days, “>30” if the patient was readmitted in more than 30 days, and “No” for no record of readmission.	0%

**Table 2:** Values of the primary diagnosis in the final dataset. In the analysis, groups that covered less than 3.5% of encounters were grouped into “other” category.

Group name	icd9 codes	Number of encounters	% of encounter	Description
Circulatory	390–459, 785	21,411	30.6%	Diseases of the circulatory system
Respiratory	460–519, 786	9,490	13.6%	Diseases of the respiratory system
Digestive	520–579, 787	6,485	9.3%	Diseases of the digestive system
Diabetes	250.xx	5,747	8.2%	Diabetes mellitus
Injury	800–999	4,697	6.7%	Injury and poisoning
Musculoskeletal	710–739	4,076	5.8%	Diseases of the musculoskeletal system and connective tissue
Genitourinary	580–629, 788	3,435	4.9%	Diseases of the genitourinary system
Neoplasms	140–239	2,536	3.6%	Neoplasms
Other (17.3%)	780, 781, 784, 790–799	2,136	3.1%	Other symptoms, signs, and ill-defined conditions
	240–279, without 250	1,851	2.6%	Endocrine, nutritional, and metabolic diseases and immunity disorders, without diabetes
	680–709, 782	1,846	2.6%	Diseases of the skin and subcutaneous tissue
	001–139	1,683	2.4%	Infectious and parasitic diseases
	290–319	1,544	2.2%	Mental disorders
	E–V	918	1.3%	External causes of injury and supplemental classification
	280–289	652	0.9%	Diseases of the blood and blood-forming organs
	320–359	634	0.9%	Diseases of the nervous system
	630–679	586	0.8%	Complications of pregnancy, childbirth, and the puerperium
	360–389	216	0.3%	Diseases of the sense organs
	740–759	41	0.1%	Congenital anomalies

**Table 3:** Distribution of variable values and readmissions (population size is 69,984).

Variable	Number of encounters	% of the population	Readmitted	
			Number of encounters	% in group
HbA1c				
No test was performed	57,080	81.6%	5,342	9.4%
Result was high and the diabetic medication was changed	4,071	5.8%	361	8.9%
Result was high but the diabetic medication was not changed	2,196	3.1%	166	7.6%
Normal result of the test	6,637	9.5%	590	8.9%
Gender				
Female	37,234	53.2%	3,462	9.3%
Male	32,750	46.8%	2,997	9.2%
Discharge disposition				
Discharged to home	44,339	63.4%	3,184	7.2%
Otherwise	25,645	36.6%	3,275	12.8%
Admission source				
Admitted from emergency room	37,277	53.3%	3,563	9.6%
Admitted because of physician/clinic referral	22,800	32.6%	2,032	8.9%
Otherwise	9,907	14.2%	846	8.5%
Specialty of the admitting physician				
Internal Medicine	10,642	15.2%	1,044	9.8%
Cardiology	4,213	6.0%	309	7.3%
Surgery	3,541	5.1%	284	8.0%
Family/general practice	4,984	7.1%	492	9.9%
Missing or unknown	33,641	48.1%	3,237	9.6%
Other	12,963	18.5%	1,093	8.4%
Primary diagnosis				
A disease of the circulatory system (icd9: 390–459, 785)	21,411	30.6%	2,129	9.9%
Diabetes (icd9: 250.xx)	5,747	8.2%	529	9.2%
A disease of the respiratory system (icd9: 460–519, 786)	9,490	13.6%	710	7.5%
Diseases of the digestive system (icd9: 520–579, 787)	6,485	9.3%	532	8.2%
Injury and poisoning (icd9: 800–999)	4,697	6.7%	524	11.2%
Diseases of the musculoskeletal system and connective tissue (icd9: 710–739)	4,076	5.8%	354	8.7%
Diseases of the genitourinary system (icd9: 580–629, 788)	3,435	4.9%	313	9.1%
Neoplasms (icd9: 140–239)	2,536	3.6%	239	9.4%
Other	12,107	17.3%	1,129	9.3%
Race				
African American	12,626	18.0%	1,116	8.8%
Caucasian	52,300	74.7%	4,943	9.5%
Other	3,138	4.5%	256	8.2%
Missing	1,920	2.7%	144	7.5%
Age^a^				
30 years old or younger	1,808	2.6%	112	6.2%
30–60 years old	21,871	31.3%	1,614	7.4%
Older than 60	46,305	66.2%	4,733	10.2%

Age (numeric)	mean	median	1st Qu.	3rd Qu.

Age in years	64.9	67	55	77
Time in hospital				
Days between admission and discharge (1–14)	4.3	3	2	6

^a^After the preliminary analysis of the relationship between age and the logistic transformation of the readmission rate. See Figure 2.

**Table 4:** Coefficients of noninteraction terms estimated from the final logistic regression model.

		Estimate	*P* value
	Intercept*	−3.180	<2*e* − 16

Discharge	Home	Reference	
	Other	0.302	0.119

Race	African American	Reference	
	Caucasian	0.015	0.760
	Missing	−0.335	0.012
	Other*	−0.267	0.009

Admission	Emergency	Reference	
	Other*	−0.155	<0.001
	referral	−0.020	0.517

Medical specialty	Cardiology	Reference	
	General practice	0.388	0.035
	Internal medicine	0.377	0.022
	Missing*	0.463	0.002
	Other	0.306	0.059
	Surgery	0.443	0.032

Time in hospital*		0.130	0.000

Age	[30, 60)	reference	
	[60, 100)	0.286	0.041
	<30	1.833	0.031

Diagnosis	Diabetes	reference	
	Circulatory	0.143	0.171
	Digestive	−0.066	0.604
	Genitourinary	−0.288	0.056
	Injury	0.022	0.878
	Musculoskeletal*	−0.627	0.000
	Neoplasms	0.146	0.375
	Other	0.065	0.558
	Respiratory	−0.299	0.013

HbA1c	Not measured	reference	
	High, changed*	−0.398	0.004
	High, not changed*	−0.579	0.009
	Normal	0.003	0.982

“Diagnosis” stands for a primary diagnosis with possible values: “circulatory” for icd9: 390–459, 785, “digestive” for icd9: 520–579, 787, “genitourinary” for icd9: 580–629, 788, “diabetes” for icd9: 250.xx, “injury” for icd9: 800–999, “musculoskeletal” for icd9: 710–739, “neoplasms” for icd9: 140–239, “respiratory” for icd9: 460–519, 786, and “other” for otherwise.

“HbA1c” variable has four values: “not measured” when the test was not measured, “normal” if the test was measured and the result was normal, “high, changed” when the result of HbA1c test was high and diabetic mediations were changed, and “high, not changed” when the result of HbA1c test was high but diabetic mediations were not changed.

*Coefficients significant at the 0.01 significance level.

**Table 5:** Coefficients of the interaction terms estimated from the final logistic regression model.

Attribute name	Value	Attribute name	Value	Estimate	*P*-value
Age	[60, 100)		General Practice	0.061	0.732
			Internal Medicine	−0.018	0.910
			Missing	−0.112	0.446
			Other	−0.127	0.423
		Medical specialty	Surgery	−0.202	0.306
	<30		General Practice	−2.465	0.013
			Internal Medicine	−1.980	0.028
			Missing	−1.490	0.083
			Other*	−2.419	0.006
			Surgery	−2.715	0.041

Diagnosis	Circulatory	Discharge	Other	−0.073	0.510
	Digestive			−0.004	0.980
	Genitourinary			−0.188	0.235
	Injury			0.253	0.086
	Musculoskelet			0.325	0.057
	Neoplasms			−0.137	0.435
	Other			0.182	0.124
	Respiratory			0.079	0.540

Race	Caucasian	Discharge	Other	0.030	0.678
	Missing			0.320	0.087
	Other*			0.514	<0.001

Discharge		Time in hospital*		−0.030	0.001

Medical Specialty	General Practice	Discharge	Other	0.340	0.057
	Internal Medicine			0.211	0.199
	Missing			0.237	0.121
	Other			0.391	0.018
	Surgery*			0.733	0.000

Time in hospital		Medical Specialty	General Practice	−0.0591	0.023
			Internal Medicine	−0.0357	0.121
			Missing*	−0.0575	0.007
			Other	−0.0517	0.027
			Surgery*	−0.1179	0.000

Age	[60, 100)		General Practice	0.061	0.732
			Internal Medicine	−0.018	0.910
			Missing	−0.112	0.446
			Other	−0.127	0.423
		Medical specialty	Surgery	−0.202	0.306
	<30		General Practice	−2.465	0.013
			Internal Medicine	−1.980	0.028
			Missing	−1.490	0.083
			Other*	−2.419	0.006
			Surgery	−2.715	0.041

Time in hospital		Diagnosis	Circulatory	−0.036	0.032
			Digestive	−0.032	0.144
			Genitourinary	0.043	0.084
			Injury	−0.043	0.056
			Musculoskelet	0.020	0.457
			Neoplasms	−0.047	0.071
			Other*	−0.060	<0.001
			Respiratory	−0.009	0.651

	High, changed	Diagnosis	Circulatory^∗a^	0.573	<0.001
			Digestive	0.487	0.092
			Genitourinary	0.428	0.164
			Injury	−0.183	0.612
			Musculoskelet	0.754	0.037
			Neoplasms	0.122	0.806
			Other	0.305	0.129
			Respiratory^b^	0.313	0.175
HbA1c	High, not changed		Circulatory^a^	0.578	0.024
			Digestive	0.215	0.616
			Genitourinary	0.000	1.000
			Injury	0.316	0.517
			Musculoskelet	0.799	0.132
			Neoplasms	1.046	0.075
			Other	0.646	0.029
			Respiratory^b^	0.435	0.191
	Normal		Circulatory^a^	−0.066	0.694
			Digestive	0.010	0.965
			Genitourinary	0.189	0.441
			Injury	−0.595	0.020
			Musculoskelet	−0.100	0.719
			Neoplasms	0.362	0.215
			Other	−0.060	0.742
			Respiratory^b^	−0.484	0.021

“Diagnosis” stands for a primary diagnosis with possible values: “circulatory” for icd9: 390–459, 785, “digestive”—icd9: 520–579, 787; “genitourinary”—icd9: 580–629, 788, “diabetes”—icd9: 250.xx, “injury” icd9: 800–999, “musculoskeletal”—icd9: 710–739; “neoplasms”—icd9: 140–239,“ respiratory” icd9: 460–519, 786, and “other” otherwise.

“HbA1c” variable has four values: “Not measured”, when the test was not measured, “Normal” if the test was measured and the result was normal, “High, changed” when the result of HbA1c test was high and diabetic mediations were changed, and “High, not changed” when the result of HbA1c test was high but diabetic mediations were not changed.

^a^Denotes *P* value less than 0.001 for a three degree of freedom test.

^b^Denotes *P* value equal to 0.02 for a three degree of freedom test.

*Denotes coefficients significant at the 0.01 significance level.

## References

[1] Umpierrez GE, Isaacs SD, Bazargan N, You X, Thaler LM, Kitabchi AE (2002). Hyperglycemia: an independent marker of in-hospital mortality in patients with undiagnosed diabetes. *Journal of Clinical Endocrinology and Metabolism*.

[2] Levetan CS, Passaro M, Jablonski K, Kass M, Ratner RE (1998). Unrecognized diabetes among hospitalized patients. *Diabetes Care*.

[3] Siegelaar SE, Hoekstra JBL, Devries JH (2011). Special considerations for the diabetic patient in the ICU; targets for treatment and risks of hypoglycaemia. *Best Practice and Research: Clinical Endocrinology and Metabolism*.

[4] Pittas AG, Siegel RD, Lau J (2004). Insulin therapy for critically ill hospitalized patients: a meta-analysis of randomized controlled trials. *Archives of Internal Medicine*.

[5] Tricco AC, Ivers NM, Grimshaw JM (2012). Effectiveness of quality improvement strategies on the management of diabetes: a systematic review and meta-analysis. *The Lancet*.

[6] Lansang MC, Umpierrez GE (2008). Management of inpatient hyperglycemia in noncritically ill patients. *Diabetes Spectrum*.

[7] Vinik R, Clements J (2011). Management of the hyperglycemic inpatient: tips, tools, and protocols for the clinician. *Hospital Practice*.

[8] Cios KJ, Moore GW (2002). Uniqueness of medical data mining. *Artificial Intelligence in Medicine*.

[9] Frank A, Asuncion A (2010). *UCI Machine Learning Repository*.

[10] Bergenstal RM, Fahrbach JL, Iorga SR, Fan Y, Foster SA (2012). Preadmission glycemic control and changes to diabetes mellitus treatment regimen after hospitalization. *Endocrine Practice*.

[11] Baldwin D, Villanueva G, McNutt R, Bhatnagar S (2005). Eliminating inpatient sliding-scale insulin: a reeducation project with medical house staff. *Diabetes Care*.

[12] Anwar H, Fischbacher CM, Leese GP (2011). Assessment of the under-reporting of diabetes in hospital admission data: a study from the Scottish diabetes research network epidemiology group. *Diabetic Medicine*.

[13] Geiss LS, Pan L, Cadwell B, Gregg EW, Benjamin SM, Engelgau MM (2006). Changes in Incidence of Diabetes in U.S. Adults, 1997–2003. *The American Journal of Preventive Medicine*.

[14] Shah RV, Altman RK, Park MY (2012). Usefulness of hemoglobin A(1c) to predict outcome after cardiac resynchronization therapy in patients with diabetes mellitus and heart failure. *The American Journal of Cardiology*.

[15] Halkos ME, Puskas JD, Lattouf OM (2008). Elevated preoperative hemoglobin A1c level is predictive of adverse events after coronary artery bypass surgery. *Journal of Thoracic and Cardiovascular Surgery*.

[16] Britton KA, Aggarwal V, Chen AY (2011). No association between hemoglobin A1c and in-hospital mortality in patients with diabetes and acute myocardial infarction. *The American Heart Journal*.

[17] Moghissi ES, Korytkowski MT, DiNardo M (2009). American association of clinical endocrinologists and American diabetes association consensus statement on inpatient glycemic control. *Diabetes Care*.

[18] Stolker JM, Spertus JA, McGuire DK (2012). Relationship between glycosylated hemoglobin assessment and glucose therapy intensification in patients with diabetes hospitalized for acute myocardial infarction. *Diabetes Care*.

